# Response: Commentary: Single-Cell Sequencing Analysis and Weighted CoExpression Network Analysis Based on Public Databases Identified That TNC Is a Novel Biomarker for Keloid

**DOI:** 10.3389/fimmu.2022.923283

**Published:** 2022-06-28

**Authors:** Jiaheng Xie, Yuan Cao, Liang Chen, Ming Wang

**Affiliations:** ^1^ Department of Burn and Plastic Surgery, The First Affiliated Hospital of Nanjing Medical University, Jiangsu Province Hospital, Nanjing, China; ^2^ Fourth School of Clinical Medicine, Nanjing Medical University, Nanjing, China; ^3^ Department of General Surgery, Fuyang Hospital Affiliated to Anhui Medical University, Fuyang, China

**Keywords:** keloid, immune microenvironment, inflammation, competitive endogenous RNA, bioinformatics

## Introduction

Keloid is an abnormal healing process of the wound, which is often secondary to the traumatic site, surgical incision, and even the needle path (1–3). Due to its rapid proliferation and growth rate, the keloid often protrudes from the skin and extends beyond the wound edge (4). This makes keloid a nasty disease that seriously affects their daily life and psychology (5). Histologically, the keloid is characterized by significant overformation of collagen fibers (6). It is worth mentioning that the immune microenvironment, previously thought to play an important role in tumors, is also believed to be involved in the formation and development of keloid in recent years (7). Multiple types of immune cells are speculated to constitute the chronic inflammatory background of keloid, and the inflammatory factors are involved in the activation and maintenance of keloid growth pathways (8). However, the keloid’s immune microenvironment is not well understood.

In recent years, advances in bioinformatics techniques have made it possible to explore keloid genomics, proteomics, and the immune microenvironment in depth. In our previous paper “Single-Cell Sequencing Analysis and Weighted Co-Expression Network Analysis Based on Public Databases Identified That TNC Is a Novel Biomarker for Keloid” which was published on 22 December 2021 in Frontiers in Immunology, the genomics of keloid was explored to a certain extent and tenascin-c(TNC) was identified as a robust marker of keloid by single-cell sequencing analysis and WGCNA (9). This article was commented by Xia et al. on Apr 04, 2022 (10). We are pleased to see that Xia et al. have added co-expression analysis, enrichment analysis, and correlation analysis of epithelial mesenchymal transformation(EMT)of TNC in keloid in that commentary article (10). The author’s research can provide some reference for the role of TNC in keloid. However, the gene regulation mechanism of TNC in keloid and its role in immune microenvironment remain unclear.

**Figure 1:**
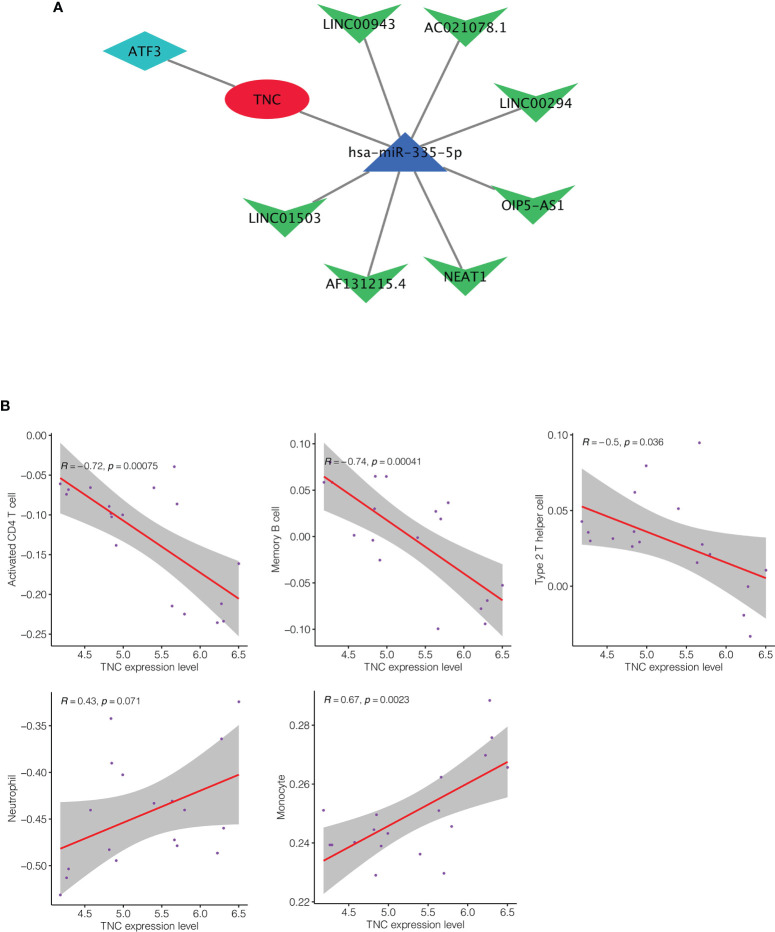
**(A)** The competitive endogenous RNA (ceRNA) regulatory network of TNC in keloid. **(B)** Immune correlation analysis of TNC in keloid. TNC had a significant negative correlation with CD4+ T cells, memory B cells and Type 2 T helper cells in keloid. And TNC showed significant positive correlation with neutrophils and monocytes in keloid.

In this response, we further explored the regulatory mechanism of TNC in keloid and its role in the immune microenvironment. First, we constructed the TNC’s competitive endogenous RNA (ceRNA) regulatory network in keloid by Mirtarbase and Starbase (**Figure 1A**). In this ceRNA network, we can see the interaction between TNC and transcription factors and non-coding RNAs. Subsequent immune correlation analysis showed that TNC had a significant negative correlation with CD4+ T cells, memory B cells and Type 2 T helper cells in keloid (**Figure 1B**). However, TNC showed significant positive correlation with neutrophils and monocytes in keloid (**Figure 1B**). This provides some reference for us to understand the role of TNC in the inflammatory background of keloid.

Tenascin-c (TNC), as an extracellular matrix protein, plays an important role in the maintenance of normal physiological functions and various pathological processes. In recent years, TNC has been widely studied for its important role in wound healing and the pathogenesis of keloid. In 1999, Dalkowski et al. found that TNC was highly expressed in keloid compared with normal tissues (11). That same year, Yamada found that Tocoretinate inhibited the contraction of collagen matrix in human fibroblasts with high TNC expression (12). Our previous study identified TNC as the hub gene in the pathogenesis of keloid through single-cell sequencing analysis and weighted coexpression analysis. These evidences all suggest that TNC may be a potential target for keloid therapy in the future. More studies are needed in the future to explore the significance of TNC in keloid.

## Author Contributions

JX and LC designed the study. LC was involved in database search and statistical analyses. LC and JX were involved in the writing of manuscript and its critical revision. MW and JX was responsible for the submission of the final version of the paper. All authors approved the final version. All authors agree to be accountable for all aspects of the work.

## Conflict of Interest

The authors declare that the research was conducted in the absence of any commercial or financial relationships that could be construed as a potential conflict of interest.

## Publisher’s Note

All claims expressed in this article are solely those of the authors and do not necessarily represent those of their affiliated organizations, or those of the publisher, the editors and the reviewers. Any product that may be evaluated in this article, or claim that may be made by its manufacturer, is not guaranteed or endorsed by the publisher.
